# The Role of the EZH2 and H3K27me3 Expression as a Predictor of Clinical Outcomes in Salivary Duct Carcinoma Patients: A Large-Series Study With Emphasis on the Relevance to the Combined Androgen Blockade and HER2-Targeted Therapy

**DOI:** 10.3389/fonc.2021.779882

**Published:** 2022-02-03

**Authors:** Natsuki Saigusa, Hideaki Hirai, Yuichiro Tada, Daisuke Kawakita, Masato Nakaguro, Kiyoaki Tsukahara, Satoshi Kano, Hiroyuki Ozawa, Takahito Kondo, Kenji Okami, Takafumi Togashi, Yukiko Sato, Makoto Urano, Manami Kajiwara, Tomotaka Shimura, Chihiro Fushimi, Akira Shimizu, Isaku Okamoto, Takuro Okada, Takayoshi Suzuki, Yorihisa Imanishi, Yoshihiro Watanabe, Akihiro Sakai, Koji Ebisumoto, Yuichiro Sato, Yoshitaka Honma, Keisuke Yamazaki, Yushi Ueki, Toyoyuki Hanazawa, Yuki Saito, Hideaki Takahashi, Mizuo Ando, Shinji Kohsaka, Takashi Matsuki, Toshitaka Nagao

**Affiliations:** ^1^ Department of Anatomic Pathology, Tokyo Medical University, Tokyo, Japan; ^2^ Department of Head and Neck Oncology and Surgery, International University of Health and Welfare, Mita Hospital, Tokyo, Japan; ^3^ Department of Otorhinolaryngology, Head and Neck Surgery, Nagoya City University Graduate School of Medical Sciences, Nagoya, Japan; ^4^ Department of Pathology and Laboratory Medicine, Nagoya University Hospital, Nagoya, Japan; ^5^ Department of Otorhinolaryngology, Head and Neck Surgery, Tokyo Medical University, Tokyo, Japan; ^6^ Department of Otolaryngology Head and Neck Surgery, Faculty of Medicine and Graduate School of Medicine, Hokkaido University, Sapporo, Japan; ^7^ Department of Otorhinolaryngology Head and Neck Surgery, Keio University School of Medicine, Tokyo, Japan; ^8^ Department of Otorhinolaryngology, Head and Neck Surgery, Tokyo Medical University Hachioji Medical Center, Tokyo, Japan; ^9^ Department of Otolaryngology Head and Neck Surgery, Tokai University School of Medicine, Isehara, Japan; ^10^ Department of Head and Neck Surgery, Niigata Cancer Center Hospital, Niigata, Japan; ^11^ Department of Pathology, Cancer Institute Hospital, Japanese Foundation for Cancer Research, Tokyo, Japan; ^12^ Department of Diagnostic Pathology Bantane Hospital Fujita Health University, School of Medicine, Nagoya, Japan; ^13^ Department of Otolaryngology, Showa University Fujigaoka Hospital, Yokohama, Japan; ^14^ Department of Head and Neck, Esophageal Medical Oncology, National Cancer Center Hospital, Tokyo, Japan; ^15^ Department of Otolaryngology Head and Neck Surgery, Niigata University Graduate School of Medical and Dental Sciences, Niigata, Japan; ^16^ Department of Otolaryngology, Head and Neck Surgery, Chiba University Graduate School of Medicine, Chiba, Japan; ^17^ Department of Otolaryngology - Head and Neck Surgery, Faculty of Medicine, The University of Tokyo, Tokyo, Japan; ^18^ Department of Otorhinolaryngology, Head and Neck Surgery, Yokohama City University, School of Medicine, Yokohama, Japan; ^19^ Department of Otolaryngology-Head & Neck Surgery, Okayama University Graduate School of Medicine, Dentistry and Pharmaceutical Sciences, Okayama, Japan; ^20^ Division of Cellular Signaling, National Cancer Center Research Institute, Tokyo, Japan; ^21^ Department of Otorhinolaryngology, Head and Neck Surgery, Kitasato University School of Medicine, Kanagawa, Japan

**Keywords:** salivary duct carcinoma, EZH2, H3K27me3, androgen receptor, HER2, combined androgen blockade (CAB), prognosis, therapeutic effect

## Abstract

**Objective:**

Salivary duct carcinoma (SDC) is a highly aggressive and uncommon tumor arising not only *de novo* but also in pleomorphic adenoma. Androgen receptor (AR)- and HER2-targeted therapy have recently been introduced for SDC as promising treatment options; however, no predictive biomarkers have yet been established. EZH2 and H3K27me3 are closely linked to the development and progression of various cancers, and EZH2 is also expected to be a desirable therapeutic target. We therefore explored the clinicopathological and prognostic implications of EZH2 and H3K27me3 in a large cohort of SDC patients, focusing on their impact on the therapeutic efficacy of AR- or HER2-targeted therapy.

**Materials and Methods:**

The EZH2 and H3K27me3 immunohistochemical expression and *EZH2* Y646 gain-of-function mutation status were examined in 226 SDCs, and the relationship with the clinicopathological factors as well as clinical outcomes were evaluated within the three groups depending on the treatment: AR-targeted (combined androgen blockade with leuprorelin acetate and bicalutamide; 89 cases), HER2-targeted (trastuzumab and docetaxel; 42 cases), and conventional therapy (112 cases).

**Results:**

EZH2 and H3K27me3 were variably immunoreactive in most SDCs. A positive correlation was found between the expression of EZH2 and H3K27me3. The EZH2 expression in the SDC component was significantly higher than that in the pre-existing pleomorphic adenoma component. *EZH2* Y646 was not identified in any cases. EZH2-high cases more frequently had an advanced clinical stage and aggressive histological features than EZH2-low cases. An EZH2-high status in patients treated with AR-targeted therapy was associated with a significantly shorter progression-free and overall survival as well as a lower objective response rate and clinical benefit rate. In addition, a H3K27me3-high status in patients treated with AR-targeted therapy was related to a shorter overall survival. Conversely, there was no association between the EZH2 and H3K27me3 expression and the clinical outcomes in the conventional or HER2-targeted therapy groups.

**Conclusions:**

A high expression of EZH2 and H3K27me3 in SDC might be a predictor of a poor efficacy of AR-targeted therapy. Our data provide new insights into the role of EZH2 and H3K27me3 in therapeutic strategies for SDC.

## Introduction

Salivary duct carcinoma (SDC) is a highly aggressive and uncommon tumor that accounts for as many as 10% of all salivary gland malignancies (1, 2). It can occur not only as *de novo* carcinoma but also as a malignant component of carcinoma ex pleomorphic adenoma (PA) (1, 3). SDC is histologically comparable to high-grade mammary ductal carcinoma. SDC shows a high rate of metastasis, and systemic chemotherapy is required for patients with metastatic disease.

Most SDCs characteristically express androgen receptor (AR), and approximately 40% are positive for HER2 (4–6). Recently, based on these biomarker profiles, treatments targeting AR and HER2 have been developed as a promising optional therapy in recurrent/metastatic or unresectable locally advanced SDCs (7–14). AR-targeted therapy demonstrated equivalent efficacy and less toxicity for patients with AR-positive SDC than conventional chemotherapy (9, 10, 14, 15). Furthermore, HER2-targeted therapy showed more encouraging efficacy with a higher response rate in HER2-positive SDC patients than conventional or AR-targeted therapy (8–11). However, since SDCs often express both AR and HER2, selecting the most appropriate treatment remains difficult.

In the past decade, there have been remarkable advances in research on therapy-relevant biomarkers linked to biological behavior in various cancers. At present, little is known concerning the mechanisms and factors related to resistance to targeted therapy in patients with SDC, although a few possible adverse biomarkers of SDC patients treated with AR-targeted therapy, such as AR-related molecules, have been reported (3, 16–19). However, how to apply such strategies in clinical practice remains challenging (19). For this reason, precise immunohistochemical biomarkers that reflect the clinicopathological status or predict the prognosis and therapeutic effect are awaited (3–6, 19).

Enhancer of zeste homolog 2 (EZH2), a specific histone methyltransferase of histone H3 at Lys 27 (H3K27), has been garnering attention as a prognostic factor as well as an attractive target for cancer therapy. EZH2 plays an important role in the epigenetic maintenance of the repressive chromatin mark. It forms the polycomb repressive complex 2 (PRC2) and demonstrates histone methyltransferase activity (20). PRC2 recruitment to chromatin causes H3K27 trimethylation (H3K27me3), which is normally related to gene repression and plays a crucial role in tumor development (21). Furthermore, the *EZH2* Y646 gain-of-function mutation is involved in tumorigenesis (22–24). In fact, the overexpression of EZH2 has been shown to be associated with invasive growth and poor clinical outcomes in many malignant tumors, including breast, prostate, gastric, endometrial and hematologic cancers, even though the prognostic impact of H3K27me3 expression is variable (25–29). Furthermore, the overexpression of EZH2 is related to resistance to AR- and HER2-targeted therapy in prostate and breast cancers, respectively (30, 31).

An EZH2 inhibitor was approved by the U.S. Food and Drug Administration for use against epithelioid sarcoma and follicular lymphoma in 2020 (32). In addition, several clinical trials concerning EZH2 inhibitor therapy for different types of malignant tumors are ongoing (ClinicalTrials.gov: NCT02601950, NCT01897571 and NCT04407741) (33, 34). To our knowledge, however, the roles of EZH2 and H3K27me3 in SDC have not yet been described.

We therefore examined the EZH2 and H3K27me3 protein expression and *EZH2* Y646 activating mutations and evaluated their relationship with the clinicopathological factors and prognosis of SDC in a large cohort of patients. Furthermore, we sought to analyze the association of EZH2/H3K27me3 expression with survival outcomes and therapeutic effect within differently (AR- or HER2-targeted) treated groups of patients with SDC.

## Materials and Methods

This study was approved by the Institutional Ethics Review Board of each participating institution.

### Patients

**Figure 1:**
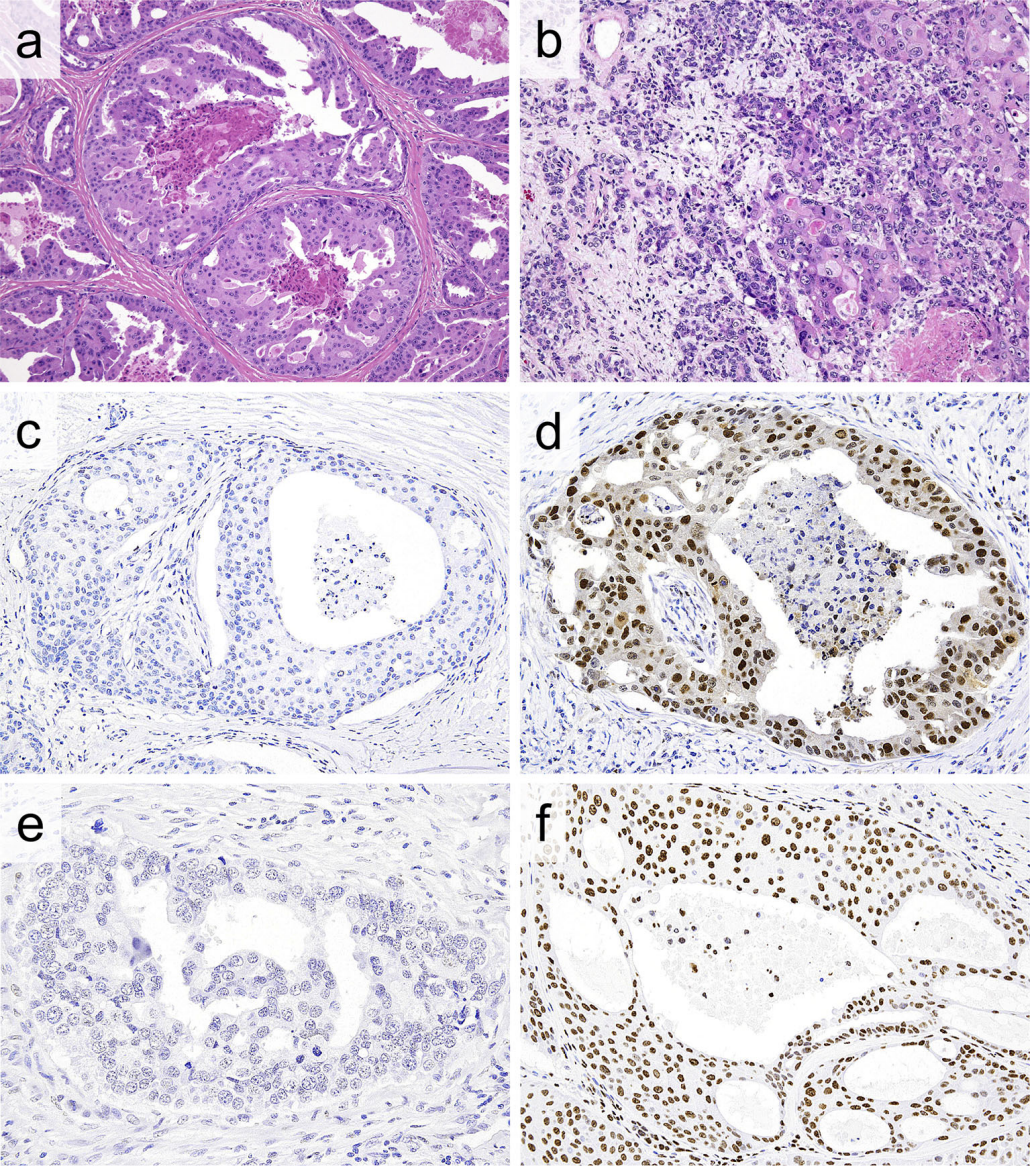
**(A, B)** Representative histologic features of salivary duct carcinoma (SDC). **(A)** Dilated ductal structures with a papillary, “Roman-bridge,” or cribriform growth accompanied by comedo necrosis. **(B)** SDC ex pleomorphic adenoma composed of SDC (right portion) and a preexisting pleomorphic adenoma component (left portion). Note carcinoma cells exhibiting large pleomorphic nuclei and abundant eosinophilic cytoplasm. **(C, D)** Immunohistochemically, the EZH2 labelling index (LI) is low (0%) **(C)** and high (90%) **(D)** in SDC. **(D)** Diffuse and strong nuclear and weak cytoplasmic EZH2 immunoreactivity. **(E, F)** Likewise, the H3K27me3 LI is low (0%) **(E)** and high (90%) **(F)** in SDC. **(F)** Diffuse and strong nuclear H3K27me3 immunoreactivity.

**Figure 2:**
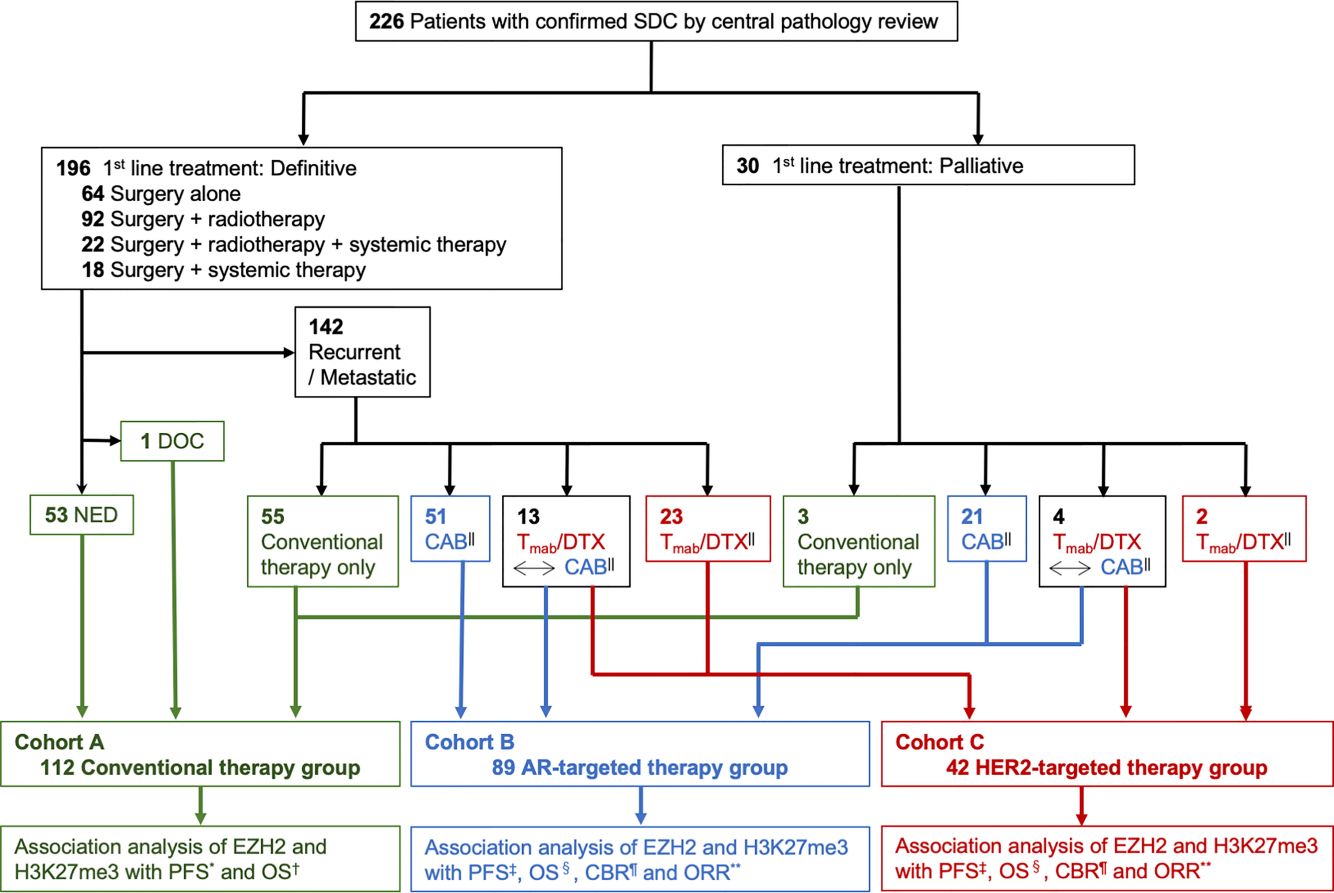
Study flow diagram. SDC, salivary duct carcinoma; DOC, dead of other cause; NED, no evidence of disease; Tmab/DTX, trastuzumab and docetaxel; CAB, combined androgen blockade; AR, androgen receptor; HER2, human epidermal growth factor receptor type 2; PFS, progression free survival; OS, overall survival; CBR, clinical benefit rate; ORR, objective response rate. * Time from the start of any treatment to the diagnosis of progressive disease. ^†^ Time from the start of any treatment to death from any cause or the last follow-up. ^‡^ Time from the start of AR- or HER2-targeted therapy to the diagnosis of progressive disease or death from any cause. ^§^ Time from the start of AR- or HER2-targeted therapy to death from any cause or the last follow-up. || ± conventional therapy. ^¶^ The percentage of patients who achieved a complete response (CR), partial response (PR) or stable disease for at least 24 weeks. ** The percentage of patients who achieved CR or PR.

All patients underwent a central pathological review by an expert pathologist (T.N.) according to the rigorous histomorphological criteria for SDC (**Figure 1**). We recruited 226 patients who were diagnosed with and received treatment for SDC at 7 institutions between 1994 and 2019, and AR- and HER2-tageted therapy started in 2012 and 2011, respectively. As shown in the study flow diagram (**Figure 2**), we classified total 226 patients into 3 independent cohort groups: the conventional therapy group (Cohort A; 112 cases, 49.6%), the AR-targeted therapy group (Cohort B; 89 cases, 39.4%), and the HER2-targeted therapy group (Cohort C; 42 cases, 18.6%). The conventional therapy group (Cohort A) was defined as SDC patients who did not receive either AR-targeted therapy (combined androgen blockade therapy [CAB]: leuprorelin acetate and bicalutamide) (9) or HER2-targeted therapy (trastuzumab and docetaxel) (11–13). Patients who had been treated before the introduction of targeted therapy were also assigned to the conventional therapy group (Cohort A), even if they were positive for AR and/or HER2. Almost all patients in the conventional therapy group (Cohort A) (109 of 112 cases, 97%) received radical surgical resection with or without radiotherapy/systemic therapy, which is considered a typical treatment in general clinical practice. In addition, Cohorts B and C included 17 patients who received both AR- and HER2-targeted therapy. The details of AR- and HER2-targeted therapy were previously reported (9, 11).

We retrospectively reviewed the patient records to obtain information about the age, sex, tumor size, lymph node metastasis, distant metastasis, and survival. The TNM classification was determined in accordance with the 8th edition of the International Union Against Cancer (35).

### Histopathology

The histopathological analysis regarding tumor grading was performed using a previously reported histological risk stratification model, which was determined by 4 histological features (prominent nuclear pleomorphism, mitosis ≥30/10 high-power fields, vascular invasion and high poorly differentiated cluster) (36). The total number of positive factors was considered to indicate low risk to high risk, as follows: low risk, 0 to 1 point; intermediate risk, 2 to 3 points; high risk, 4 points.

### Immunohistochemistry (IHC) and Fluorescence *In Situ* Hybridization (FISH)

For IHC, formalin-fixed, paraffin-embedded tumor tissue was cut into 4-μm-thick sections. A polymer-based detection system with heat-mediated antigen retrieval was conducted using the primary antibodies shown in **Supplementary Table 1**. Diaminobenzidine was applied to detect antigen-antibody reactions. The EZH2 and H3K27me3 labelling index (LI) (0–100%) was determined by counting the number of immunoreactive nuclei in at least 1,000 cells (**Figure 1**). We also compared the EZH2/H3K27me3 expression among normal salivary glands, the PA component and carcinoma.

HER2 was considered to be positive based on an HER2 IHC score of 3+ and/or *HER2* amplification, as determined by a FISH analysis, in accordance with the ASCO/CAP guideline for evaluating breast cancer (5, 37). The analysis methods of immunohistochemical staining for Ki-67, AR, p53, p-Akt, mTOR, PTEN, EGFR and CK5/6 were reported previously by our group (5, 37–40)

### Gene Mutation Analyses

We extracted DNA from paraffin-embedded sections using a QIAamp DNA FFPE Tissue Kit (Qiagen, Hilden, DE, USA) and DNA was purified using a QIAquick Spin Kit (Qiagen). DNA purity was tested using a NanoDrop (Thermo Scientific, Waltham, MA, USA). For the detection of mutations, DNA was amplified with primers flanking regions in exon 16 of the *EZH2* gene encompassing codon 646. We amplified the region with the following primers: forward primer 5’-TGG GGG ATT TTT ATC AAA G-3’/reverse primer 5’-TCA AAC CCA CAG ACT TAC CT-3’. Polymerase chain reaction products were sequenced in both sense and antisense directions using a BigDye Terminator v3.1 cycle sequencing kit on an ABI 3730 instrument (Applied Biosystems, Inc., Foster City, CA, USA). Sanger sequencing was performed for *TP53* (exons 4–10), *PIK3CA* (exons 9 and 20) *and HRAS* (exons 1-2) (38).

### Statistical Analyses

Non-continuous variables were compared using the chi-squared test. Continuous variables were compared using the Mann-Whitney U test or Wilcoxon’s signed-rank test. Spearman’s rank correlation test was used to evaluate the correlation between the expression of proteins. The association between the EZH2/H3K27me3 expression and the overall survival (OS) or progression-free survival (PFS) was evaluated using the Kaplan-Meier product-limit method and univariate and multivariate Cox proportional hazards models. Furthermore, in the AR- and HER2-targeted therapy groups (Cohorts B and C), the relationship between the EZH2/H3K27me3 expression and clinical benefit rate (CBR) or objective response rate (ORR) was also analyzed using univariate and multivariate Cox proportional hazards models. The potential confounders in the multivariate analysis included the age, sex, primary tumor site, separate T, N, and M classification, first-line treatment, histological origin and AR- and HER2-targeted therapy. Conventional therapy group (Cohort A), AR-targeted therapy group (Cohort B), and HER2-targeted therapy group (Cohort C) were independent cohorts classified based on a difference of therapy, but not clinical outcomes. Therefore, we estimated the optimal cut-off values for the EZH2 and H3K27me3 expression according to survival in each cohort. The PFS in the conventional therapy group (Cohort A) was defined as the length of time from the start of any treatment to the diagnosis of progressive disease, while the OS in the conventional therapy was defined as the length of time from the start of any treatment to death from any cause. The PFS in the AR- and HER2-targeted therapy groups (Cohorts B and C) was defined as the length of time from the start of AR- and HER2-targeted therapy to the diagnosis of progressive disease or death from any cause, respectively, while the OS in the AR- and HER2-targeted therapy groups (Cohorts B and C) was defined as the length of time from the start of AR- and HER2-targeted therapy to death from any cause or the last follow-up, respectively.

The therapeutic effect of AR- and HER2-targeted therapy was evaluated according to the ORR, defined as the percentage of patients who achieved a complete response (CR) or partial response (PR) and CBR, which was defined as the percentage of patients who achieved CR, PR or stable disease (SD) for at least 24 weeks. Tumor assessments were performed within 4 weeks before the initiation of AR- and HER2-targeted therapy using computed tomography and/or magnetic resonance imaging and were repeated every 6–8 weeks until disease progression, death, or up to 2 years after the initiation of treatment. Thereafter, assessment was continued every 3 months in surviving patients. Patient response was determined based on the Response Evaluation Criteria in Solid Tumors (version 1.1) (41). All statistical analyses were performed using the STATA software program (version 16; StataCorp, College Station, TX, USA). All tests were two-sided, and *P* values of < 0.05 were considered to indicate statistical significance.

## Results

### Patients’ Characteristics

The distribution of the patient characteristics is shown in **Table 1**. The case series included 194 males and 32 females with a median age of 63 (range, 26-94) years old. Eighty-three SDC cases (43%) and 115 cases (57%) were classified as *de novo* and carcinoma ex PA, respectively (**Figure 1**). Bone-only metastasis was found in 4 cases in the AR- group (Cohort B) and 1 case in the HER2-targeted therapy group (Cohort C). In the conventional therapy group (Cohort A), 42 of 112 cases (37.5%) were treated with systemic therapy, either at the time of the initial treatment or at the time of recurrence/metastasis.

**Table 1:** Patients’ characteristics.

	Total cohort n = 226		Cohort A		Cohort B*		Cohort C*	
			Conventional therapy group n = 112		AR-targeted therapy group n = 89		HER2-targeted therapy group n = 42	
	n	%	n	%	n	%	n	%
**Age (years)**								
≤65	125	55	59	53	41	46	29	69
>65	101	45	53	47	48	54	13	31
**Sex**								
Male	194	86	95	85	81	91	32	76
Female	32	14	17	15	8	9	10	24
**Primary site**								
Parotid gland	172	76	92	82	61	69	28	67
Others	53	23	20	18	27	30	14	33
Unknown	1	1	0	0	1	1	0	0
**Histological origin**								
CXPA	115	51	42	37	37	42	5	12
*de novo*	83	37	67	60	29	33	32	76
undefined	28	12	3	3	23	25	5	12
**AR expression**								
<20%	32	14	27	24	0	0	5	12
≥20%	194	86	85	76	89	100	37	88
**HER2 status**								
Negative	131	58	62	55	67	75	2	5
Positive	95	42	50	45	22	25	40	95
**T classification**								
1	21	9	8	7	–	–	–	–
2	59	26	31	28	–	–	–	–
3	46	21	21	19	–	–	–	–
4a	91	40	49	44	–	–	–	–
4b	7	3	3	3	–	–	–	–
Unknown	2	1	0	0				
**N classification**								
0	94	42	60	54	–	–	–	–
1	15	7	9	8	–	–	–	–
2	114	50	43	38	–	–	–	–
3	3	1	0	0	–	–	–	–
**M classification**								
0	194	86	106	95	–	–	–	–
1	32	14	6	5	–	–	–	–
**First-line treatment**								
Surgery	196	87	109	97	–	–	–	–
Radiation	123	54	57	51	–	–	–	–
Systemic therapy	65	29	23	21	–	–	–	–

CXPA, carcinoma ex pleomorphic adenoma; AR, androgen receptor; HER2, human epidermal growth factor receptor type 2. *Cohorts B and C included 17 patients who received both AR- and HER2-targeted therapy.

The median follow-up period of all patients was 3.7 (range 0.04-19.0) years. The 5-year OS rate in all patients was 46.9% (95% confidence interval [CI] 39.8%-53.7%), and the 5-year PFS rate was 23.5% (95% CI 18.0%-29.4%). The median OS of all patients was 4.4 (95% CI 3.7-5.9) years, and the median PFS was 1.0 (95% CI 0.9-1.3) years. In addition, the median follow-up period of conventional therapy group (Cohort A) was 4.0 (range 0.04-19.0) years. The median OS of conventional therapy group (Cohort A) was 5.8 (95% CI 3.4-8.7) years, and the median PFS was 2.6 years (95% CI not significant).

### Efficacy of AR-Targeted Therapy

The median follow-up period in the AR-targeted therapy group (Cohort B) was 1.9 (range 0.1-6.6) years. The responses in patients treated with CAB are shown by waterfall plots in **Supplementary Figure 1**. Four (4.5%), 20 (22.5%), 42 (47.2%), and 23 (25.8%) patients showed CR, PR, SD, and PD, respectively. The ORR was 27.0% (95% CI 18.7%–37.2%). Forty-two patients with SD maintained their status for more than 24 weeks and CBR was 74.2% (95% CI 63.9–82.3%). The median PFS was 0.46 (95% CI 0.36–0.58) years, and the median OS was 2.33 (95% CI 1.86-3.17) years.

### Efficacy of HER2-Targeted Therapy

The median follow-up period in the HER2-targeted therapy group (Cohort C) was 2.3 (range 0.3-8.4) years. The responses in patients treated with HER2-targeted therapy are shown by waterfall plots in **Supplementary Figure 1**. Five (12.2%), 22 (53.7%), 11 (26.8%) and 3 (7.3%) patients showed CR, PR, SD and PD, respectively. The ORR was 65.9% (95% CI 49.8%–79.0%). Eleven patients with SD maintained the status for more than 24 weeks and CBR was 92.7% (95% CI 79.0%–97.7%). The median PFS was 0.80 (95% CI 0.56–0.93) years, and the median OS was 2.91 (95% CI 2.27-3.27) years.

### The Expression of EZH2 and H3K27me3 With Clinicopathological Correlation

In virtually all cases, both EZH2 and H3K27me3 were expressed in at least a limited part of the SDC (97.8% and 99.1%, respectively). The cut-off values for a low/high LI of EZH2 and H3K27me3 were 60% and 65%, respectively, based on the median value. A total of 124 cases (54.9%) and 102 cases (45.1%) were thus classified into the EZH2-low and EZH2-high groups, respectively (mean EZH2 expression LI: 48.8%). Likewise, 112 cases (52.6%) and 101 cases (47.4%) were categorized into the H3K27me3-low and H3K27me3-high groups, respectively (mean H3K27me3 expression LI: 52.8%) (**Figure 1**). A weak positive correlation was found between the expression of EZH2 and H3K27me3 (*r* = 0.357, *P* < 0.001) (**Supplementary Figure 2**).

The EZH2 expression of the surrounding non-neoplastic salivary gland tissues and pre-existing PA components was very low (mean EZH2 expression LI: 1.8% and 4.2%, respectively), and the value in the SDC was significantly higher than that in the PA component (*P* < 0.001), while that in the PA component was higher than that in normal salivary gland tissue (*P* = 0.002) (**Supplementary Figure 3**). In contrast, H3K27me3 expression was also observed in the surrounding non-neoplastic salivary gland tissues and pre-existing PA components to varying degrees in almost all cases (mean H3K27me3 expression LI: 39.1% and 52.0%, respectively). The expression of H3K27me3 in the PA component and SDC was higher than that in the normal salivary gland tissue (*P* = 0.038 and < 0.001, respectively); however, the H3K27me3 expression in the PA component and SDC was not significantly associated (*P* = 0.885) (**Supplementary Figure 3**).

The correlations between the EZH2/H3K27me3 expression and the clinicopathological factors and various biomarkers are summarized in **Table 2** and **Supplementary Table 2**. High-EZH2-LI cases more frequently had an advanced N and M classification compared with low-EZH2-LI cases (*P* = 0.005 and < 0.001, respectively), while there was no notable relationship between the EZH2 expression and T classification. In addition, an EZH2-high tumor was associated with the presence of prominent nuclear pleomorphism, intermediate or high histological risk group, carcinoma ex PA, higher Ki-67 LI and the aberrant expression of p53 in comparison to an EZH2-low tumor (*P* < 0.001, = 0.015, = 0.014, < 0.001 and = 0.005, respectively). In contrast, an H3K27me3-high status was associated with a low p-Akt and high EGFR expression (*P* = 0.036 and 0.034, respectively). A weak positive correlation was found between the expression of H3K27me3 and AR (*r* = 0.350, *P* < 0.001) (**Supplementary Figure 4**).

**Table 2:** Patient characteristics and the correlation between the EZH2/H3K27me3 expression and clinicopathological factors.

Clinicopathological factors		EZH2 expression			H3K27me3 expression		
		<60%	≥60%	*P*	<65%	≥65%	*P*
	n (%)	n = 124	n = 102		n = 112	n = 101	
**H3K27me3 expression, mean ± SD (%)**		46.2 ± 25.2	61.5 ± 22.4	<0.001*	NA	NA	NA
**Age, mean ± SD, years**		62.7 ± 12.7	63.4 ± 12.0	0.886	62.0 ± 13.3	63.4 ± 10.9	0.588
**Sex**							
Male	194 (86)	106	88	0.865	96	86	0.907
Female	32 (14)	18	14		16	15	
**Histologic origin**							
* De novo*	83 (43)	57	26	0.014*	41	38	0.391
CXPA	115 (57)	59	56		64	46	
**T classification**							
1-2	80 (36)	46	34	0.562	34	41	0.116
3-4	144 (64)	77	67		77	59	
**N classification**							
0	94 (42)	62	32	0.005*	45	46	0.429
1-2	132 (58)	62	70		67	55	
**M classification**							
0	194 (86)	117	77	<0.001*	101	84	0.131
1	32 (14)	7	25		11	17	
**Prominent nuclear pleomorphism**							
Absent	68 (35)	52	16	<0.001*	36	30	0.744
Present	128 (65)	67	61		69	52	
**Mitosis (/10 HPF)**							
<30	98 (50)	65	33	0.108	49	45	0.265
≥30	98 (50)	54	44		56	37	
**Lymphatic invasion**							
Absent	119 (58)	71	48	0.827	57	55	0.056
Present	86 (42)	50	36		53	29	
**Vascular invasion**							
Absent	88 (43)	52	36	0.987	47	36	0.986
Present	117 (57)	69	48		63	48	
**Perineural invasion**							
Absent	104 (51)	62	42	0.861	55	41	0.869
Present	101 (49)	59	42		55	43	
**Histologic risk stratification model†**							
Low risk	43 (22)	33	10	0.015*	22	20	0.576
Intermediate or high risk	153 (78)	86	67		83	62	
**AR expression, mean ± SD (%)**		63.3 ± 32.0	62.3 ± 31.8	0.925	55.4 ± 34.2	70.5 ± 27.7	0.001*
**HER2 status**							
Negative	131 (58)	76	55	0.264	62	62	0.373
Positive	95 (42)	48	47		50	39	
**Ki-67 LI, mean ± SD (%)**		36.9 ± 23.3	50.5 ± 20.7	<0.001*	42.8 ± 24.4	42.9 ± 21.3	0.883
**p53**							
NE	127 (56)	80	47	0.005*	60	60	0.391
EN/EP	99 (44)	44	55		52	41	
***TP53***							
Wild-type	64 (35)	42	22	0.111	25	34	0.106
Mutation	118 (65)	63	55		62	50	

EZH2, enhancer of zeste homologue 2; H3K27me3, histone H3 trimethylation at lysine 27; SD, standard deviation; NA, not available; CXPA, carcinoma ex pleomorphic adenoma; HPF, high-power fields; AR, androgen receptor; HER2, human epidermal growth factor receptor type 2; LI, labeling index; NE, not extreme; EN/EP, extreme negative/positive. †The histologic risk stratification model was determined by 4 histologic features (prominent nuclear pleomorphism, mitosis ≥30/10 HPF, vascular invasion, and high PDC). The total number of positive factors among these 4 was defined as indicating low to high risk as follows: low risk, 0 to 1 point; intermediate risk, 2 to 3 points; high risk, 4 points. *Statistically significant association (P < 0.05).

### Association Between the EZH2/H3K27me3 Expression and Clinical Outcomes

We estimated the optimal cut-off values based on survival in each cohort (Cohorts A-C). Consequently, cut-off values between the low and high LI of EZH2 and H3K27me3 in conventional therapy group (Cohort A) were determined to be 35% and 50%, respectively. These in AR-targeted therapy group (Cohort B) were 60% and 80%, respectively. Furthermore, these in HER2-targeted therapy group (Cohort C) were determined to be 65% and 70%, respectively.

**Figure 3:**
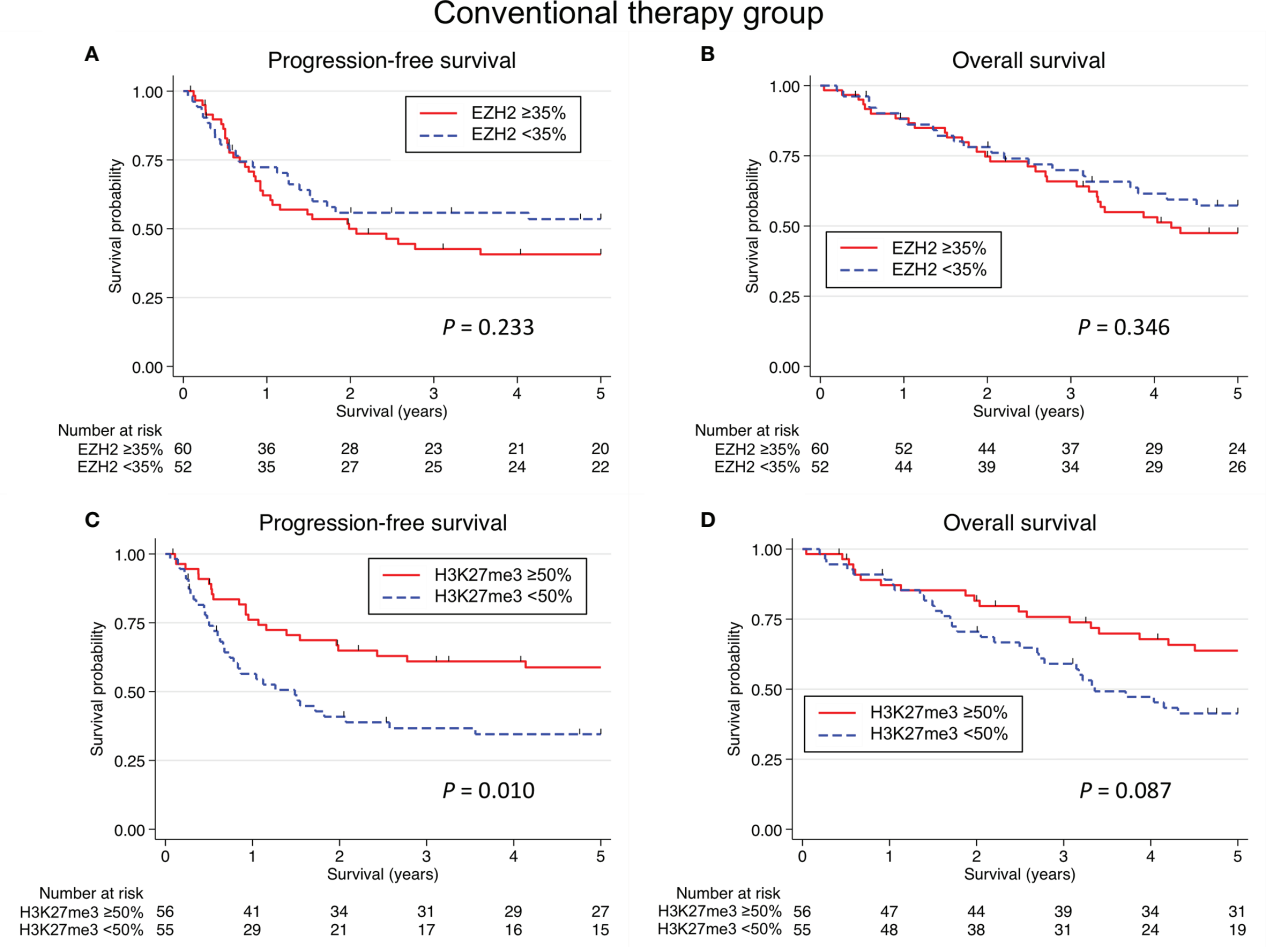
Kaplan-Meier survival curves according to the EZH2/H3K27me3 expression in salivary duct carcinoma with conventional therapy. No significant association was identified between the EZH2 expression and the progression-free survival (PFS) **(A)** or overall survival (OS) **(B)**. **(C)** A low expression of H3K27me3 was associated with a significantly shorter PFS (*P* = 0.010), but there was no significant association between the H3K27me3 expression and the PFS in multivariate analysis. **(D)** Although a low expression of H3K27me3 tented to be associated with a shorter OS (*P* = 0.087), no significant relation between the H3K27me3 expression and the OS was detected in univariate and multivariate analyses.

In the conventional therapy group (Cohort A), although the high expression of H3K27me3 was associated with a significantly longer PFS only in the univariate analysis (*P* = 0.011), there were no other significant prognostic associations (**Table 3** and **Figure 3**).

**Table 3:** The association between EZH2 or H3K27me3 expression and clinical outcomes in patients with salivary duct carcinoma treated with conventional therapy.

		Progression-free survival						Overall survival					
		Univariate analysis			Multivariate analysis			Univariate analysis			Multivariate analysis		
	n	HR	95% CI	*P*	HR	95% CI	*P*	HR	95% CI	*P*	HR	95% CI	*P*
**EZH2 expression**													
≥35%	60	1.00	–	–	1.00	–	–	1.00	–	–	1.00	–	–
<35%	52	0.73	0.43-1.23	0.235	0.69	0.38-1.24	0.210	0.78	0.47-1.30	0.347	0.70	0.39-1.28	0.247
**H3K27me3 expression**													
≥50%	56	1.00	–	–	1.00	–	–	1.00	–	–	1.00	–	–
<50%	55	1.99	1.17-3.39	0.011*	1.51	0.77-2.96	0.227	1.56	0.94-2.59	0.086	0.97	0.52-1.80	0.924

Adjusted by age, sex, primary tumor site, TNM classification, first-line treatment, and histologic origin.

HR, hazard ratio; CI, confidence interval; EZH2, enhancer of zeste homologue 2; H3K27me3, histone H3 trimethylation at lysine 27.

*Statistically significant association (P<0.05).

**Figure 4:**
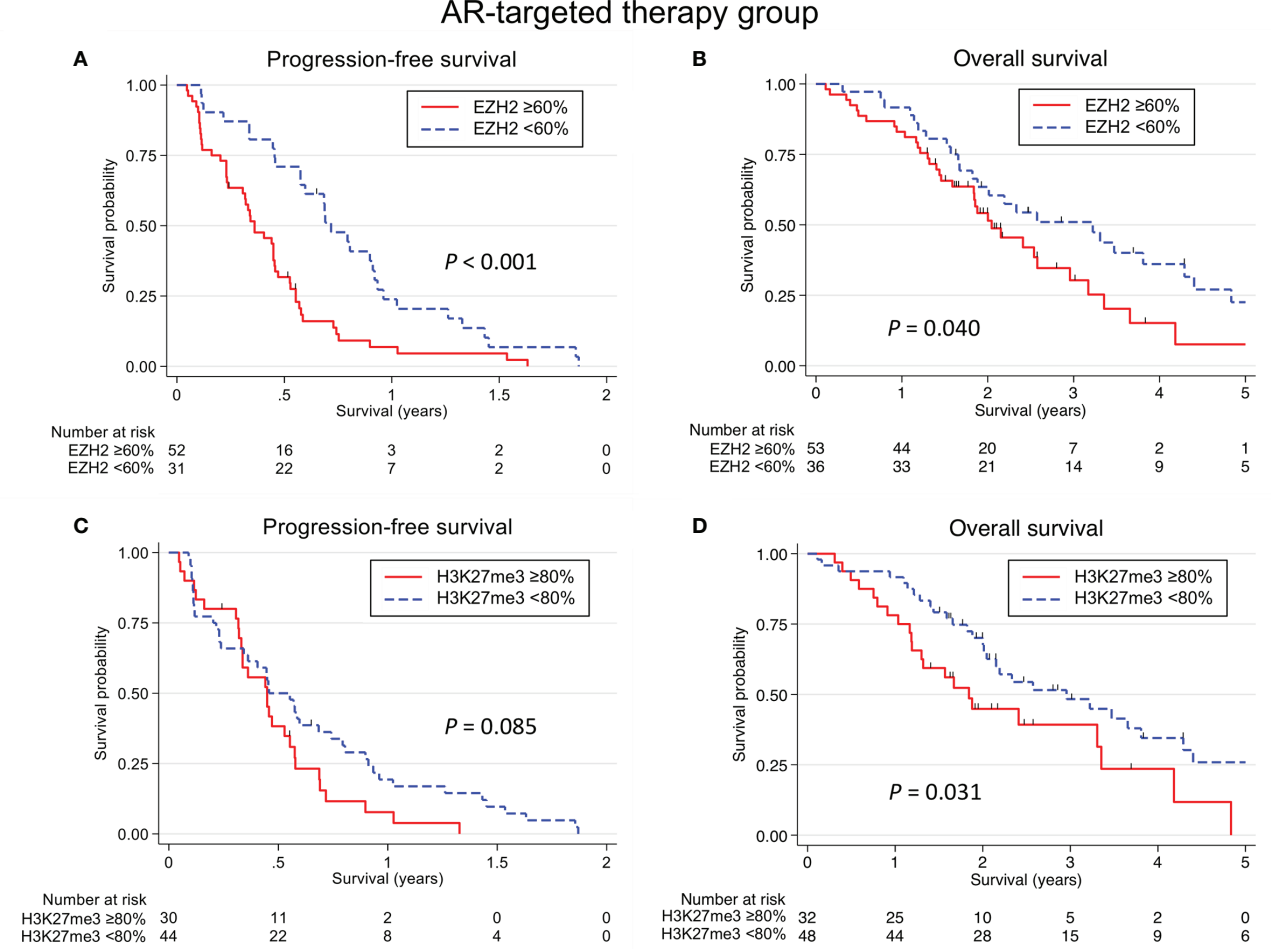
Kaplan-Meier survival curves according to the EZH2/H3K27me3 expression in salivary duct carcinoma treated with AR-targeted therapy. An EZH2-high status was associated with a significantly shorter progression-free survival (PFS) **(A)** and overall survival (OS) **(B)** (*P <*0.001 and *P* = 0.040, respectively). **(C)** There was no significant association between the H3K27me3 expression and the PFS. **(D)** An H3K27me3-high status was associated with a shorter OS (*P* = 0.031).

**Figure 5:**
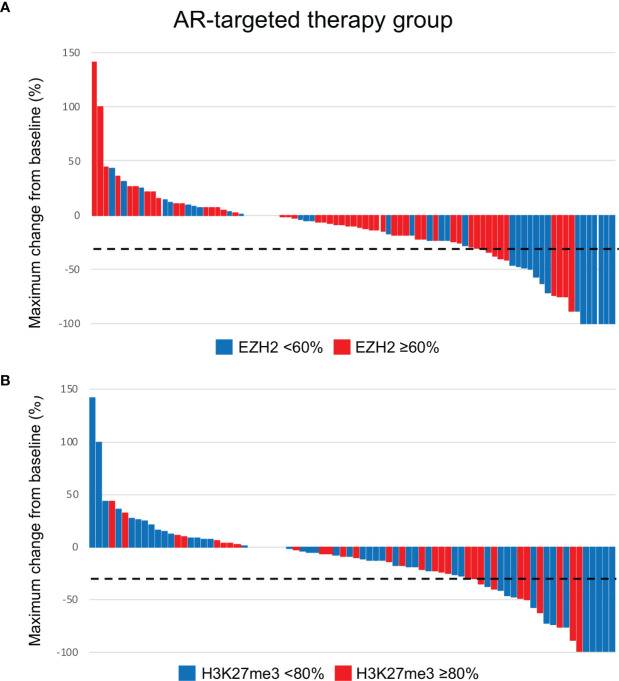
Waterfall plots of maximum changes from baseline according to the EZH2 **(A)** and H3K27me3 **(B)** status in patients who received AR-targeted therapy. The dotted line indicates -30% of maximum change from baseline.

In the AR-targeted therapy group (Cohort B), univariate and multivariate analyses revealed that an EZH2-high status was associated with a significantly shorter PFS (*P* < 0.001) (**Table 4** and **Figure 4**). A significant relationship between an EZH2-high status and a shorter OS was identified in the univariate analysis (*P* = 0.042), but not in the multivariate analysis. Furthermore, an EZH2-high status was associated with reduced ORR and CBR values in the univariate (*P* = 0.003 and 0.002, respectively) and multivariate analyses (*P* = 0.039 and 0.007, respectively). Furthermore, an H3K27me3-high status was associated with a shorter OS in the univariate and multivariate analyses (*P* = 0.027 and 0.047, respectively). There was no significant association between the H3K27me3 expression and the PFS, ORR or CBR. Waterfall plots of the maximum tumor size change from baseline according to EZH2 and H3K27me3 status are shown in **Figure 5**.

**Table 4:** The association between EZH2 or H3K27me3 expression and clinical outcomes in patients with salivary duct carcinoma treated with AR- or HER2-targeted therapy.

	AR-targeted therapy												
Variable			PFS					OS					
				Univariate analysis		Multivariate analysis			Univariate analysis			Multivariate analysis	
	n	%	median (months; 95% CI)	HR (95% CI)	*P*	HR (95% CI)	*P*	median (months; 95% CI)	HR (95% CI)	*P*		HR (95% CI)	*P*
**EZH2 expression**													
*≥60%	53	60	4.4 (2.9-5.5)	1.00	–	1.00	–	24.9 (19.3-36.0)	1.00	–		1.00	–
<60%	36	40	8.7 (7.0-11.2)	0.42 (0.26-0.68)	<0.001*	0.18 (0.09-0.36)	<0.001*	39.2 (22.2-52.2)	0.57 (0.33-0.98)	0.042*		0.53 (0.27-1.03)	0.060
**H3K27me3 expression**													
≥80%	32	40	5.5 (3.9-6.7)	1.00	–	1.00	–	22.4 (14.4-40.8)	1.00	–		1.00	–
<80%	48	60	5.6 (2.9-9.0)	0.63 (0.38-1.04)	0.070	0.56 (0.29-1.08)	0.081	36.0 (24.5-52.2)	0.53 (0.30-0.93)	0.027*		0.46 (0.21-0.99)	0.047*
			ORR					CBR					
				Univariate analysis		Multivariate analysis			Univariate analysis			Multivariate analysis	
			ORR % (95% CI)	OR (95% CI)	*P*	OR (95% CI)	*P*	CBR % (95% CI)	OR (95% CI)	*P*		OR (95% CI)	*P*
**EZH2 expression**													
≥60%	53	60	15.1 (7.6-27.7)	1.00	–	1.00	–	66.0 (52.1-77.7)	1.00	–		1.00	–
<60%	36	40	44.4 (28.8-61.2)	4.50 (1.66-12.22)	0.003*	15.56 (2.82-85.79)	0.002*	86.1 (70.0-94.3)	3.19 (1.06-9.60)	0.039*		7.81 (1.75-34.88)	0.007*
**H3K27me3 expression**													
≥80%	32	40	25.0 (12.7-43.4)	1.00	–	1.00	–	84.4 (66.7-93.6)	1.00	–		1.00	–
<80%	48	60	31.3 (19.5-46.0)	1.36 (0.50-3.73)	0.546	2.05 (0.49-8.56)	0.327	66.7 (51.9-78.7)	0.37 (0.12-1.14)	0.084		0.32 (0.08-1.31)	0.113
			HER2-Targeted Therapy										
Variable			PFS					OS					
				Univariate analysis		Multivariate analysis			Univariate analysis			Multivariate analysis	
	n	%	median (months; 95% CI)	HR (95% CI)	*P*	HR (95% CI)	*P*	median (months; 95% CI)	HR (95% CI)	*P*		HR (95% CI)	*P*
**EZH2 expression**													
≥65%	21	50	9.8 (5.9-13.8)	1.00	–	1.00	–	30.3 (13.8-39.7)	1.00	–		1.00	–
<65%	21	50	9.7 (6.3-11.3)	1.13 (0.56-2.27)	0.730	1.30 (0.57-2.94)	0.534	35.7 (16.3-61.3)	0.74 (0.34-1.60)	0.450		0.51 (0.18-1.46)	0.211
**H3K27me3 expression**													
≥70%	18	47	9.8 (6.6-11.9)	1.00	–	1.00	–	35.7 (NS)	1.00	–		1.00	–
<70%	20	63	9.7 (5.3-13.1)	1.13 (0.55-2.34)	0.743	1.38 (0.45-4.30)	0.573	35.4 (12.2-49.4)	1.47 (0.64-3.36)	0.367		1.31 (0.38-4.49)	0.662
			ORR					CBR					
				Univariate analysis		Multivariate analysis			Univariate analysis			Multivariate analysis	
			ORR % (95% CI)	OR (95% CI)	*P*	OR (95% CI)	*P*	CBR % (95% CI)	OR (95% CI)	*P*		OR (95% CI)	*P*
**EZH2 expression**													
≥65%	21	50	66.7 (43.2-84.0)	1.00	–	1.00	–	95.2 (70.2-99.4)	1.00	–		1.00	–
<65%	21	50	65.0 (41.0-83.2)	0.93 (0.26-3.38)	0.910	0.49 (0.08-3.00)	0.442	90.0 (65.4-97.7)	0.45 (0.04-5.39)	0.529		NS	–
**H3K27me3 expression**													
≥70%	18	47	77.8 (51.4-92.0)	1.00	–	1.00	–	94.4 (66.0-99.3)	1.00	–		1.00	–
<70%	20	63	57.9 (34.1-78.5)	0.39 (0.09-1.65)	0.202	0.66 (0.09-4.74)	0.676	94.7 (67.5-99.4)	1.06 (0.06-18.30)	0.969		NS	–

Adjustment by age, sex, primary tumor site, TNM classification, first-line treatment, histological origin, AR-targeted therapy (in HER2-targeted therapy group), HER2-targeted therapy (in AR-targeted therapy group).

AR, androgen receptor; HER2, human epidermal growth factor receptor type 2; PFS, progression-free survival; OS, overall survival; CBR, clinical benefit rate (complete response + partial response + stable disease ≥24 weeks); ORR, objective response rate (complete response + partial response); HR, hazard ratio; CI, confidence interval; EZH2, enhancer of zeste homologue 2; H3K27me3, histone H3 trimethylation at lysine 27.

**Figure 6:**
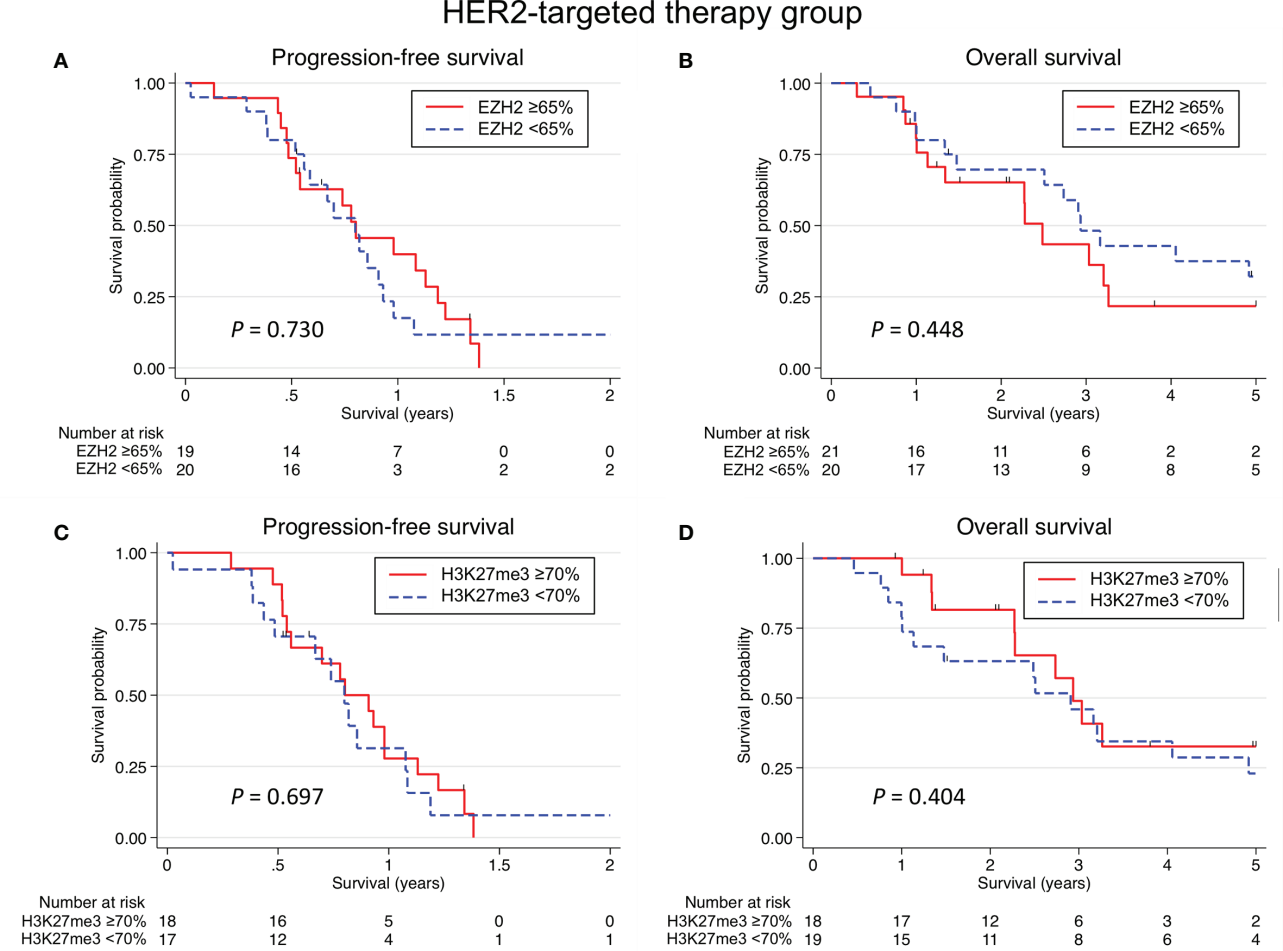
Kaplan-Meier survival curves according to the EZH2/H3K27me3 expression in salivary duct carcinoma treated with HER2-targeted therapy. No significant association was identified between the EZH2 expression and the progression-free survival (PFS) **(A)** or overall survival (OS) **(B)**. There was also no significant association between the H3K27me3 expression and the PFS **(C)** or OS **(D)**.

**Figure 7:**
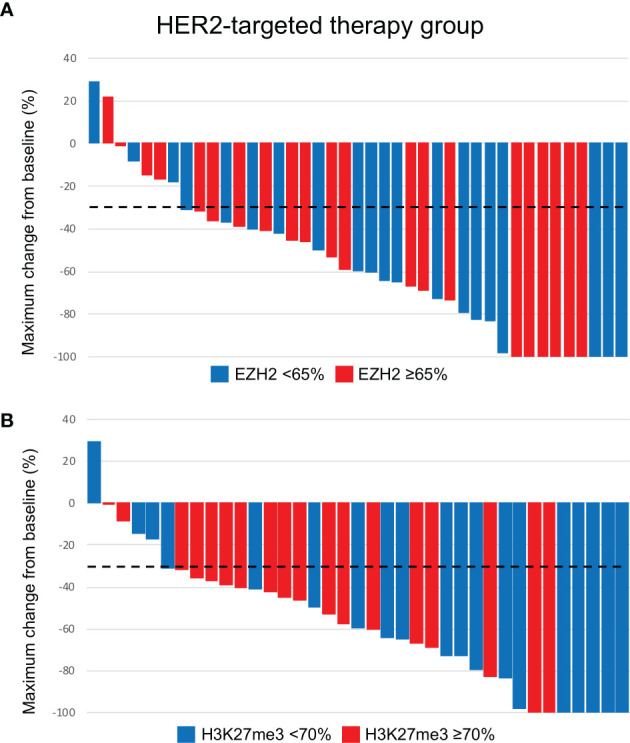
Waterfall plots of maximum changes from baseline according to the EZH2 **(A)** and H3K27me3 **(B)** status in patients who received HER2-targeted therapy. The dotted line indicates -30% of maximum change from baseline.

In contrast, no significant association was identified between the EZH2/H3K27me3 expression and therapeutic effect in the HER2-targeted therapy group (Cohort C) (**Table 4** and **Figures 6**, **7**).

### *EZH2* Y646 Activating Mutations

Two hundred and twenty-two of the 226 cases were available for gene sequencing. There were no patients with *EZH2* Y646 gain-of-function mutations.

## Discussion

The present findings suggested that the EZH2 and H3K27me3 expression was a predictive factor of AR-targeted therapy in SDC (42). Conversely, there was no significant association between the EZH2/H3K27me3 expression and clinical outcomes in the conventional or HER2-targeted therapy group.

In prostate cancer, the activated EHZ2 pathway is associated with resistance to AR-targeted therapy. First, this is because the overexpression of EZH2 promotes neuroendocrine differentiation and resistance to AR-targeted therapy through ataxia telangiectasia-mutated (ATM) upregulation (43, 44). Although this relationship was not investigated in this study, we are greatly interested in investigating this issue by reviewing recurrent/metastatic SDC cases with resistance to AR-targeted therapy as a future challenge. Targeting EZH2 represents a way of restoring AR signaling in neuroendocrine-differentiated tumor cells (44, 45). Second, EZH2 directly binds to the promoter of prostate-specific antigen, an AR-targeted gene, and inhibits its expression in CAB-resistant prostatic cancer cells (46). Third, EZH2 activates *AR* gene transcription through direct occupancy at its promoter (47). Therefore, there is the possibility that combination treatment targeting EZH2 and AR is an effective novel therapeutic regimen for the treatment of castration-resistant prostate cancer (CRPC) (46). The use of the EZH2 inhibitor tazemetostat in combination with AR-targeted therapy is currently being evaluated for its safety in CRPC (NCT04179864) (44).

In this study, the high-EZH2 expression was associated with a significantly shorter PFS and indicated a predictive factor of a poor efficacy of AR-targeted therapy assessed by ORR and CBR. The present findings suggest that SDC patients with EZH2-high status may be unsuitable for AR-targeted therapy. Combination treatment targeting EZH2 and AR might overcome resistance of AR-targeted therapy in SDC patients. Validation *via* prospective clinical trials is warranted in order to improve therapy selection and develop treatment strategies tailored for SDC patients.

In SDC patients with AR-targeted therapy, the H3K27me3 expression was not a predictive factor, but it was significantly associated with the OS. EZH2 is supposed to promote tumor progression in both an H3K27me3-dependent and H3K27me3-independent manner in cases of malignant tumor (22). Regarding the H3K27me3-dependent function, EZH2 catalyzes H3K27me3, which mediates chromatin compaction and results in the transcriptional repression of downstream genes, including tumor suppressor genes (22, 48). In contrast, as H3K27me3-independent functions, EZH2 not only promotes the methylation of non-histone proteins but also acts as a co-activator for transcription factors. These activities contribute to transcriptional suppression and co-activation (49, 50). Because the expression of EZH2 and H3K27me3 showed a weakly positive correlation in this study, the aggressiveness of SDC may be—at least partially—related to the H3K27me3-dependent function of EZH2.

In breast cancer, EZH2 activity is reported to be correlated with resistance to HER2-targeted therapy (31). However, for the present cohort of SDC patients treated with HER2-targeted therapy, as with conventional therapy, there was no association between the EZH2/H3K27me3 expression and therapeutic effect. On the other hand, we are also interested in the efficacy of certain drugs (*e.g.* trastuzumab deruxtecan) in the low-HER2 expression tumors, even in SDC (51).

An EZH2-high status was associated with aggressive clinicopathological features, including advanced N and M classification, the presence of prominent nuclear pleomorphism, intermediate or high histological risk group, a high Ki-67 LI and the aberrant expression of p53. Similar to the current findings on SDC, in various cancers, the association between the expression of EZH2 and tumor progression has been indicated (25–29). In salivary gland tumors, although the amount of data is very limited, adenoid cystic carcinoma with a high EZH2 expression showed a high Ki-67 LI (52). SDC cases with the high expression of EZH2 exhibited various aggressive clinicopathological features, but there was no significant association with the survival of patients in the conventional therapy group. One of the reasons that caused the discrepancy may be a difference in the patient population that was analyzed: all patients in **Table 2** and the conventional therapy group in **Table 3**. However, further studies are warranted to clarify the role of EZH2 in the regulation of biological behavior of the tumor. The *EZH2* Y646 gain-of-function mutation was not identified in SDC, in contrast to reports of its presence in lymphoma (22–24).

In colon cancer, EZH2 but not H3K27me3 expression is associated with progression from adenoma to carcinoma (53, 54). One previous report found that the majority of malignant salivary gland tumors, such as mucoepidermoid carcinoma and adenoid cystic carcinoma, showed positive EZH2 immunoreactivity, but all the investigated benign tumors, including PA, were negative (55), although no SDC cases were included in that study. In our cohort, nearly all cases with an SDC component of carcinoma ex PA expressed EZH2, whereas the PA component showed almost no expression of EZH2. In line with these findings for colon cancer, EZH2 may contribute to the malignant transformation from PA to SDC. 　

In our study, the ORR, median PFS, and median OS were 27.0%, 0.46 years, and 2.33 years, respectively, in the anti-androgen therapy group (n=89). On the other hand, according to the two European cohorts reported (n=34 and n=17) in the relevant literature, the outcomes varied: the ORR, median PFS, and median OS were 17.7-64.7%, 0.33-0.91 years, 1.41-3.66 years, respectively (10, 14). Thus, the outcomes of patients who received anti-androgen therapy do not necessarily seem considerably poor in comparison to the European cohorts. However, the discrepancy may be due differences in the cohort size, patient characteristics, regimens, and method of survival assessment.

In this study, we thought that it was not appropriate to apply common pre-set cut-offs when analyzing independent cohort groups. Because this is the first investigation to examine the EZH2 and H3K27me3 expression in SDC, there are no known optimal cut-off values for the EZH2 and H3K27me3 expression for any subject (e.g., clinicopathological factors in total cases or clinical outcomes in different therapeutic cohorts). Also, the biological behavior of each cohort (Cohorts A to C) varies in the present study. Therefore, in this study, to investigate the clinicopathological correlation of the EZH2 and H3K27me3 expression in the total cases we used the median values as the cutoff values. Alternatively, in Cohorts A to C, we individually estimated cut-off values according to survival. Due to the relatively small sample size in each group, internal validation was not conducted in this study. The most suitable cut-off values for the EZH2 and H3K27me3 expression should be reevaluated in a much larger series in future studies.

Several limitations associated with the present study warrant mention. First, the nonrandomized and retrospective design may have introduced bias into the data collection. Second, in this study, functional analyses of EZH2 and H3K27me3 were not performed, and could not find out details of subcellular molecular mechanisms. Further comprehensive studies, including a clinical trial, *in vitro* cell culture and patient-derived xenograft experiments, are needed to clarify the biological role of EZH2 and H3K27me3 in the development and progression of SDC.

In conclusion, the present study showed that EZH2 and H3K27me3 are frequently but unevenly expressed in SDC. In SDC patients treated with AR-targeted therapy, the high expression of EZH2 and H3K27me3 was a potential predictor of a poor efficacy of the treatment. In addition, there is a possibility that an EZH2-high status was associated with resistance to AR-targeted therapy.

## Data Availability Statement

The original contributions presented in the study are included in the article/**Supplementary Material**. Further inquiries can be directed to the corresponding author.

## Ethics Statement

The studies involving human participants were reviewed and approved by The Institutional Review Board of the International University of Health and Welfare Mita Hospital (No. 5-19-58). The patients/participants provided their written informed consent to participate in this study.

## Author Contributions

All authors contributed to the study conception, study design, material preparation, and data collection. Data analysis was performed by NS, HH, YT, DK, and TN. The first draft of the manuscript was written by NS and HH, and all authors commented on previous versions of the manuscript. All authors read and approved the final manuscript.

## Funding

This work was supported by a JSPS Grant-in-Aid for Scientific Research (C) to YT (no.21K09616), TN (no. 20K07417), TM (no. 20K07597) and DK (no. 20K10508) and a Grant-in-Aid for Young Scientists to HH (no. 19K16568) and CF (no. 21K16835).

## Conflict of Interest

The authors declare that the research was conducted in the absence of any commercial or financial relationships that could be construed as a potential conflict of interest.

## Publisher’s Note

All claims expressed in this article are solely those of the authors and do not necessarily represent those of their affiliated organizations, or those of the publisher, the editors and the reviewers. Any product that may be evaluated in this article, or claim that may be made by its manufacturer, is not guaranteed or endorsed by the publisher.
