# Supplementary material for: The Role of the EZH2 and H3K27me3 Expression as a Predictor of Clinical Outcomes in Salivary Duct Carcinoma Patients: A Large-Series Study With Emphasis on the Relevance to the Combined Androgen Blockade and HER2-Targeted Therapy
Source: Front Oncol. 2022 Feb 3;11:779882. doi: 10.3389/fonc.2021.779882 (PMC8850643; doi:10.3389/fonc.2021.779882)
Supplement: Supplementary file 1 [file Presentation_1.pptx]

## Slide 1
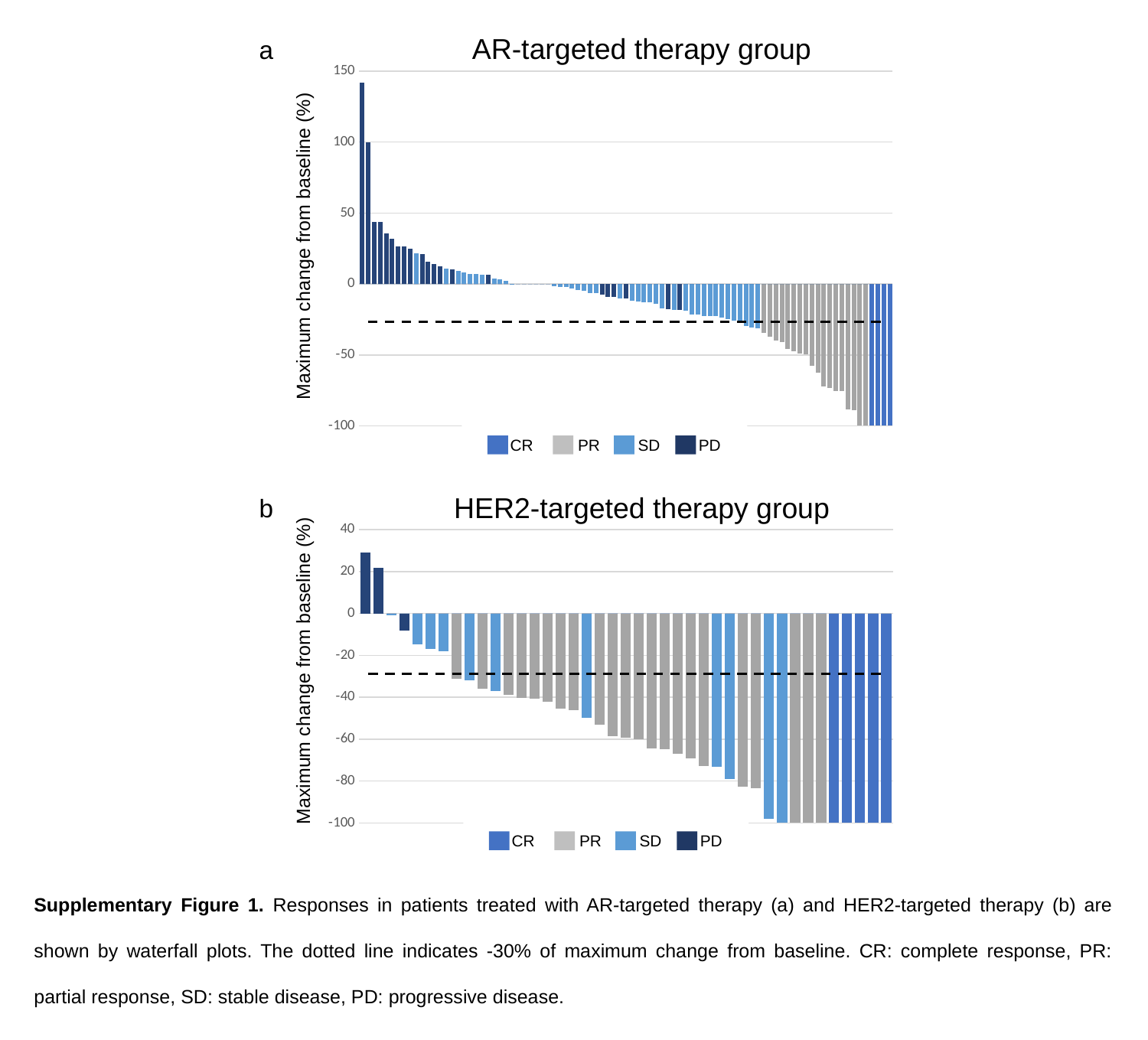

AR-targeted therapy group
a
### Chart
| Category | CR | PR | SD | PD |
|---|---|---|---|---|Maximum change from baseline (%)
CR
PR
SD
PD
HER2-targeted therapy group
b
### Chart
| Category | CR | PR | SD | PD |
|---|---|---|---|---|Maximum change from baseline (%)
CR
PR
SD
PD
Supplementary Figure 1. Responses in patients treated with AR-targeted therapy (a) and HER2-targeted therapy (b) are shown by waterfall plots. The dotted line indicates -30% of maximum change from baseline. CR: complete response, PR: partial response, SD: stable disease, PD: progressive disease.
