# Supplementary material for: The Role of the EZH2 and H3K27me3 Expression as a Predictor of Clinical Outcomes in Salivary Duct Carcinoma Patients: A Large-Series Study With Emphasis on the Relevance to the Combined Androgen Blockade and HER2-Targeted Therapy
Source: Front Oncol. 2022 Feb 3;11:779882. doi: 10.3389/fonc.2021.779882 (PMC8850643; doi:10.3389/fonc.2021.779882)
Supplement: Supplementary file 2 [file Presentation_2.pptx]

## Slide 1
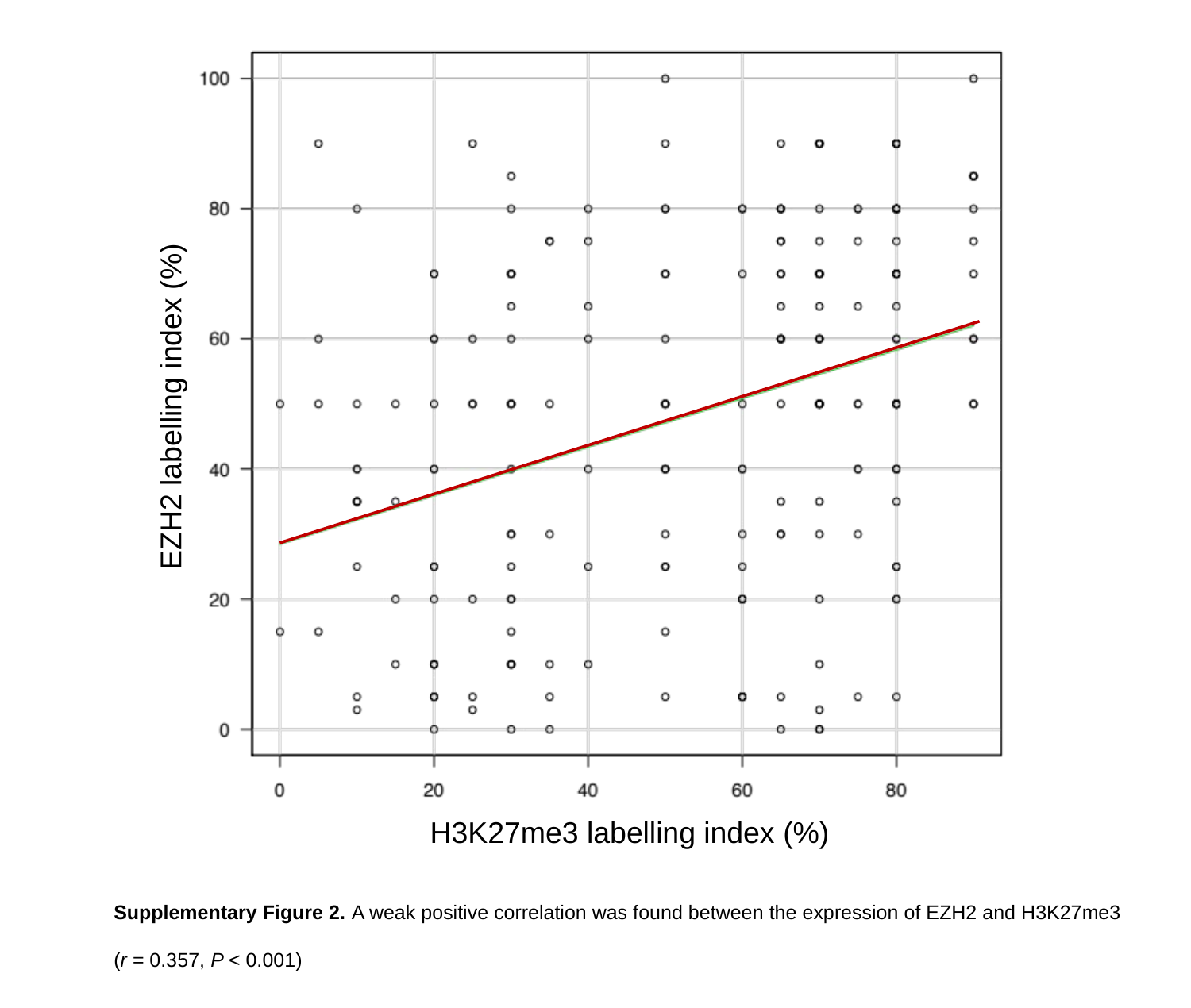

EZH2 labelling index (%)
H3K27me3 labelling index (%)
Supplementary Figure 2. A weak positive correlation was found between the expression of EZH2 and H3K27me3 (r = 0.357, P < 0.001)
