# Supplementary material for: The Role of the EZH2 and H3K27me3 Expression as a Predictor of Clinical Outcomes in Salivary Duct Carcinoma Patients: A Large-Series Study With Emphasis on the Relevance to the Combined Androgen Blockade and HER2-Targeted Therapy
Source: Front Oncol. 2022 Feb 3;11:779882. doi: 10.3389/fonc.2021.779882 (PMC8850643; doi:10.3389/fonc.2021.779882)
Supplement: Supplementary file 3 [file Presentation_3.pptx]

## Slide 1
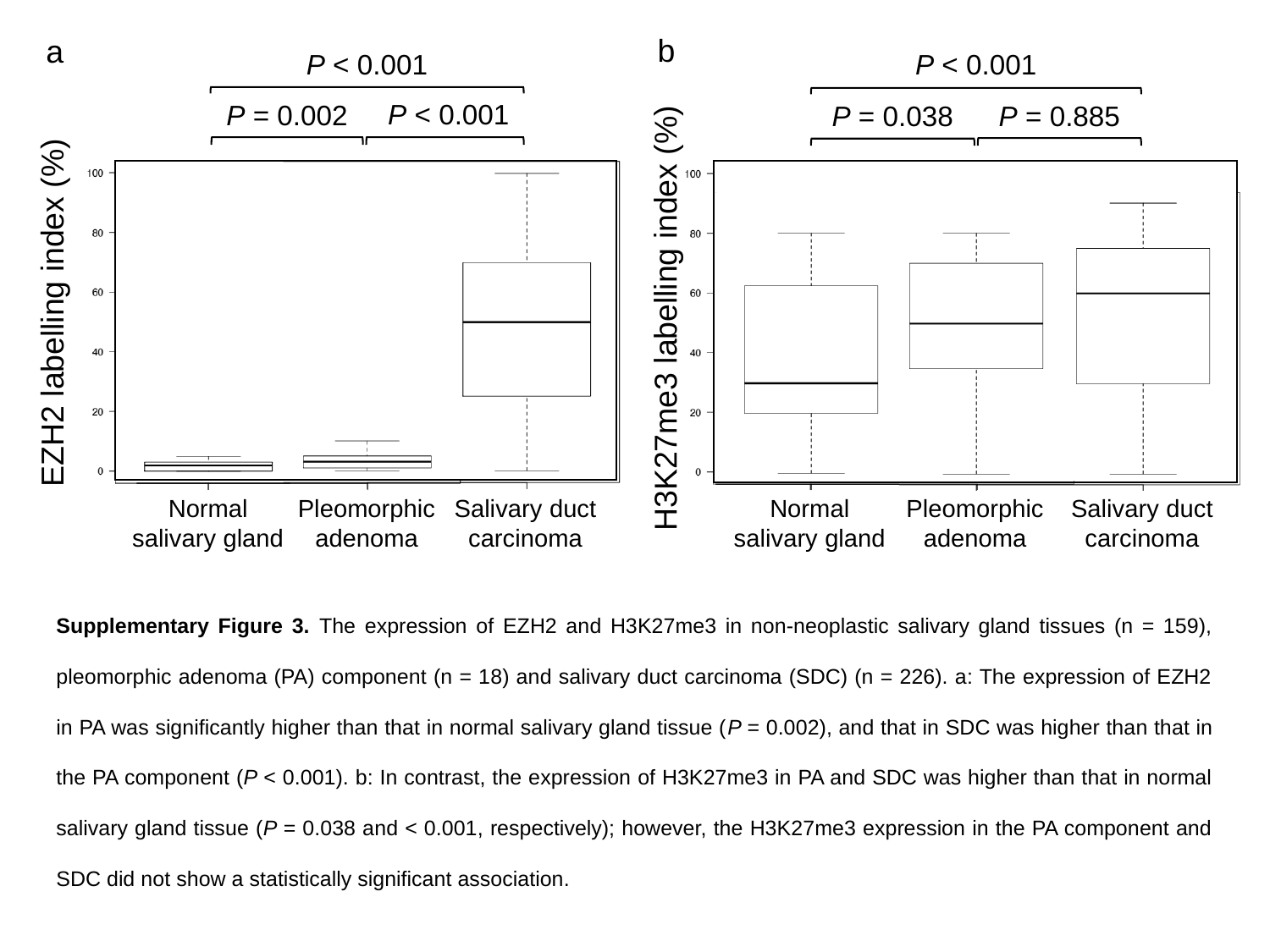

b
a
P < 0.001
P < 0.001
P < 0.001
P = 0.002
P = 0.038
P = 0.885
EZH2 labelling index (%)
H3K27me3 labelling index (%)
Normal salivary gland
Pleomorphic adenoma
Salivary duct carcinoma
Normal salivary gland
Pleomorphic adenoma
Salivary duct carcinoma
Supplementary Figure 3. The expression of EZH2 and H3K27me3 in non-neoplastic salivary gland tissues (n = 159), pleomorphic adenoma (PA) component (n = 18) and salivary duct carcinoma (SDC) (n = 226). a: The expression of EZH2 in PA was significantly higher than that in normal salivary gland tissue (P = 0.002), and that in SDC was higher than that in the PA component (P < 0.001). b: In contrast, the expression of H3K27me3 in PA and SDC was higher than that in normal salivary gland tissue (P = 0.038 and < 0.001, respectively); however, the H3K27me3 expression in the PA component and SDC did not show a statistically significant association.
